# *FCN3*可作为肺鳞癌预后及免疫治疗的潜在生物标志物

**DOI:** 10.3779/j.issn.1009-3419.2025.105.01

**Published:** 2025-03-21

**Authors:** Wei LI, Lingling ZU, Song XU

**Affiliations:** ^1^300052 天津，天津医科大学总医院肺部肿瘤外科; ^1^Department of Lung Cancer Surgery; ^2^天津市肺癌研究所，天津市肺癌转移与肿瘤微环境实验室; ^2^Tianjin Key Laboratory of Lung Cancer Metastasis and Tumor Microenvironment,Tianjin Lung Cancer Institute, Tianjin Medical University General Hospital, Tianjin 300052, China; ^3^071000 保定，河北大学附属医院胸外科; ^3^Department of Thoracic Surgery, Affiliated Hospital Hebei University, Baoding 071000, China

**Keywords:** FCN3, 肺肿瘤, 免疫, 生物标志物, FCN3, Lung neoplasms, Immunity, Biomarker

## Abstract

**背景与目的:**

非小细胞肺癌（non-small cell lung cancer, NSCLC）是全球癌症死亡的主要原因。肺鳞状细胞癌（lung squamous cell carcinoma, LUSC）是其重要的病理组织学亚型，免疫逃逸机制复杂，使得免疫治疗的效果有限。Ficolin-3（FCN3）是一种重要的免疫调节分子，它可以通过重塑肿瘤免疫微环境来调节肿瘤的免疫逃逸，但FCN3在LUSC中的作用仍不清楚。本研究使用生物信息学方法对来自癌症基因组图谱（The Cancer Genome Atlas, TCGA）数据库的LUSC样本进行了全面分析，旨在探讨FCN3在LUSC中潜在的生物学功能以及预后意义。

**方法:**

泛癌分析表征FCN3的泛癌表达特征及预后价值，同时基于TCGA数据库中的LUSC样本数据，分析FCN3在LUSC中的表达特征及预后关系。通过构建Nomogram模型、体细胞突变分析、差异分析、相关性分析及基因本体（Gene Ontology, GO）、京都基因与基因组百科全书（Kyoto Encyclopedia of Genes and Genomes, KEGG）和基因集富集分析（Gene Set Enrichment Analysis, GSEA）进一步探索FCN3的潜在作用机制。通过免疫浸润分析、免疫逃逸评分、免疫相关分子相关性分析揭示FCN3在LUSC中高表达对免疫的调节作用，同时评估FCN3特征表达与药物敏感性的相关性。体外实验验证FCN3在LUSC中的表达特征。

**结果:**

FCN3在LUSC组织中的表达水平显著低于正常组织。高表达FCN3的LUSC患者预后较差。FCN3的不同表达情况与免疫细胞浸润丰度、免疫细胞功能障碍有关联，并且与免疫检查点、免疫刺激分子、主要组织相容性复合体（major histocompatibility complex, MHC）类分子表达及化疗药物敏感性也存在关联。

**结论:**

LUSC中FCN3的高表达与不良预后有关，并且FCN3与免疫细胞浸润、相关免疫通路及免疫相关分子有关，FCN3可能是LUSC潜在的预后标志物和免疫治疗新靶点。

## Abstract (en)

**Background and objective:**

Non-small cell lung cancer (NSCLC) is the leading cause of cancer-related deaths worldwide. Lung squamous cell carcinoma (LUSC) is an important pathological subtype of NSCLC. The complex immune escape mechanism limits the effectiveness of immunotherapy. Ficolin-3 (FCN3) is a crucial immunomodulatory molecule that regulates immune escape by remodeling the tumor microenvironment. However, the role of FCN3 in LUSC remains unclear. This study employed bioinformatics methods to analyze LUSC samples from The Cancer Genome Atlas (TCGA) database. The aim of this study was to explore the potential biological functions and prognostic significance of FCN3 in LUSC.

**Methods:**

A pan-cancer analysis characterized the expression patterns and prognostic value of FCN3 across various cancer types. Simultaneously, the expression patterns of FCN3 in LUSC samples from the TCGA database and its relationship with prognosis were analyzed. The Nomogram model and somatic mutation analysis, differential expression analysis, correlation analysis, as well as Gene Ontology (GO), Kyoto Encyclopedia of Genes and Genomes (KEGG), and Gene Set Enrichment Analysis (GSEA) were constructed to explore the potential mechanisms of FCN3. Additionally, immune infiltration analysis, immune escape score (TIDE), and correlation analysis of immune-related molecules were used to reveal the regulatory role of high FCN3 levels on immunity in LUSC. Furthermore, the correlation between FCN3 expression characteristics and drug sensitivity was evaluated. Finally, in vitro experiments verified the expression characteristics of FCN3 in LUSC.

**Results:**

The expression level of FCN3 in LUSC tissues was significantly lower than that in normal tissues. Patients with high FCN3 expression in LUSC had a poorer prognosis compared to those with low expression. Different expression levels of FCN3 were associated with the abundance of immune cell infiltration and immune cell dysfunction. It was also linked to the expression of immune checkpoints, immune stimulatory molecules, major histocompatibility complex (MHC) class molecules, and chemotherapy drug sensitivity.

**Conclusion:**

High expression of FCN3 in LUSC is associated with poor prognosis and is linked to immune cell infiltration, immune-related pathways, and immune-related molecules. FCN3 may be a potential prognostic marker and a new target for immunotherapy in LUSC.

肺鳞状细胞癌（lung squamous cell carcinoma, LUSC）是非小细胞肺癌（non-small cell lung cancer, NSCLC）的一种主要亚型^[1]^。其预后差，尤其是处于晚期阶段的患者，生存率不足12%^[2]^。LUSC具有很强的化疗耐药性，导致许多患者在接受标准化疗后仍出现疾病进展，目前临床上对LUSC缺乏有效的靶向药物^[3,4]^。此外，大多数LUSC患者为驱动基因阴性，且对免疫检查点抑制剂的反应存在很大的个体差异，导致临床获益有限^[5]^，其中如纳武利尤单抗（Nivolumab）等免疫治疗药物在某些患者中显示出良好的疗效，但对于部分患者群体治疗效果仍然有限^[6]^。这可能是由于LUSC患者之间免疫微环境的异质性造成的。因此，迫切需要探寻新的用于LUSC预后及增效免疫治疗的潜在生物标志物。

Ficolin-3（FCN3）是一种重要的免疫调节因子，主要在肝脏和肺脏中表达。研究表明，FCN3在多种生物学过程中发挥着关键作用，包括免疫反应、炎症反应和细胞信号传导等。Banda等^[7]^研究表明在类风湿关节炎患者中，FCN3的表达与疾病活动性相关，提示其可能通过调节补体系统的活性，影响关节内的炎症反应。而Sun等^[8,9]^的研究表明，FCN3的缺失或功能障碍可能引起补体系统的异常激活，进而加重自身免疫反应。近期有研究^[10]^表明，FCN3的异常表达与肿瘤进展密切相关，如Zheng等^[11]^研究发现在肝细胞癌中，FCN3的表达水平与患者的生存率密切相关，能够通过激活甘露糖结合凝集素途径，参与对癌症的免疫应答，表明其在肿瘤免疫监视中的潜在作用。Michalski等^[12]^的研究显示，FCN3通过与病原体结合，促进巨噬细胞的吞噬作用，从而增强机体的免疫防御能力。此外，有研究^[13]^表明FCN3参与调节补体系统的激活，影响炎症反应的强度和持续时间，这一过程在肝细胞癌等肿瘤的发生发展中具有重要意义。另外，有研究^[14]^表明，FCN3的表达与细胞外基质成分的变化密切相关，通过调节细胞外基质的组成和结构，影响肿瘤细胞的迁移和侵袭能力。这些研究发现表明FCN3在肿瘤进展中可能具有至关重要的作用，尤其是在肿瘤免疫调节方面，同时提示FCN3可能成为癌症治疗过程中新的治疗靶点。然而，目前FCN3在LUSC中的生物学作用尚不清楚。

本研究旨在探讨LUSC中FCN3的表达与患者预后的关系。将筛选出的FCN3差异表达的关键基因和相关的枢纽基因进行功能富集分析，并研究FCN3对免疫浸润细胞丰度、免疫检查点、免疫刺激分子、主要组织相容性复合体（major histocompatibility complex, MHC）类分子及免疫微环境的影响。

## 1 资料与方法

### 1.1 常规转录组数据下载与整理

从癌症基因组图谱（The Cancer Genome Atlas, TCGA）数据库下载33种癌症的全基因组表达谱及临床数据, 特别针对LUSC（n=553，含502例肿瘤和51例正常对照），获取了TPM格式的全基因组表达谱、临床数据、拷贝数变异和单核苷酸突变数据。其中，393例有完整预后信息的肿瘤样本用于分析。

### 1.2 泛癌分析及LUSC中FCN3表达特点及预后关系

基于FCN3基因进行泛癌分析，观察这些基因在泛癌中的表达、对生存的影响、免疫浸润情况。采用使用survival包进行比例风险假设检验并进行Cox回归分析，使用ggplot2[3.4.4]进行森林图可视化计算FCN3表达对肿瘤预后的影响。最后，通过单样本基因集富集分析（Single-sample Gene Set Enrichment Analysis, ssGSEA）算法，计算所有肿瘤样本的免疫浸润评分，并探究免疫浸润评分与预后基因表达水平的相关性。用stats包调查LUSC与正常样本间FCN3表达量差异；基于人类蛋白质图谱（Human Protein Atlas, HPA）数据库，对FCN3在LUSC组织及正常肺组织中的免疫组化染色表达情况展开分析。

### 1.3 FCN3与临床病理特征相关性分析

从TCGA数据库获取LUSC的转录组测序数据及临床数据，对FCN3的表达与临床病理特征关系进行研究。

### 1.4 预后分析

应用R包survival，进行比例风险假设检验并拟合生存回归模型；结合survminer包和ggplot2包进行可视化分析。基于FCN3的中值表达，将样本分为FCN3高表达和低表达组，通过Log-rank检验，探索FCN3与LUSC患者的总生存期（overall survival, OS）、疾病特异生存期（disease specific survival, DSS）之间的关系。

### 1.5 基于FCN3表达构建Nomogram模型

根据TCGA中LUSC患者的基因表达矩阵集临床信息，首先单因素Cox风险回归筛选变量，根据多因素风险回归结果，使用R包rms建立Nomogram模型预测LUSC患者1、3、5年生存率，采用预后校准曲线和受试者工作特征（receiver operating characteristic, ROC）曲线验证效能。

### 1.6 体细胞突变分析

为了探讨LUSC中FCN3表达与体细胞突变特征的关联，我们基于FCN3基因表达的中位数将461例LUSC样本分为高表达组和低表达组。使用maftools R包对每组的体细胞突变进行分析，绘制肿瘤突变负荷（tumor mutational burden, TMB）的箱线图，并对高低表达组的TMB差异进行比较。此外，采用Oncoplot（瀑布图）展示每组的体细胞突变谱，重点比较突变负荷最大的前20个突变基因。

### 1.7 差异分析

依据FCN3基因的表达量，将LUSC的480例肿瘤样本划分为FCN3低表达组（对照组）与高表达组（实验组）。采用R包limma，并设定严格的筛选标准：差异表达的对数转换倍数（|log2Fold Change|）≥2，且经过Benjamini-Hochberg（BH）方法校正后的P<0.05。这一步骤旨在挖掘出受FCN3表达水平显著影响的基因集。

### 1.8 相关性分析与蛋白质-蛋白质互相作用（protein-protein interaction, PPI）网络

为探究与FCN3相互作用的mRNA分子，使用Spearman相关系数（r）在R环境中，借助corrplot包计算FCN3与LUSC样本中其他mRNA分子的相关性。筛选标准设为P<0.05且|r|>0.5，以识别显著且高度相关的mRNA集合。随后，使用STRING数据库进行PPI网络构建。利用Cytoscape软件进一步分析PPI网络的关键节点，识别可能在肿瘤发生和发展中起重要作用的核心基因。这为深入理解FCN3的相互作用网络提供了基础。

### 1.9 基因本体（Gene Ontology, GO）和京都基因与基因组百科全书（Kyoto Encyclopedia of Genes and Genomes, KEGG）通路富集分析

我们运用了GO富集分析和KEGG通路分析，以探究FCN3高、低表达组间差异表达基因的生物学功能和代谢通路。GO分析关注基因的生物学过程、分子功能和细胞组成，而KEGG分析则利用综合性数据库挖掘显著变化的代谢通路。采用R包clusterProfiler进行分析，设定P<0.05为显著性阈值，以揭示FCN3表达变化对基因功能和通路的影响。

### 1.10 GSEA

为了深入剖析FCN3表达数据的功能特性，并确定与特定生物学表型密切相关的基因集合，采纳R包clusterProfiler对log2Fold Change值排序的基因列表执行了GSEA。为确保分析结果的稳健性和可靠性，在每次GSEA分析中均实施了1000次的基因集置换。选用的参考基因集为c2.cp.kegg.v7.5.1.symbols，该基因集源自Molecular Signatures Database（MSigDB）数据库，涵盖了广泛的代谢途径和功能分类信息。分别针对FCN3的差异表达分子集合和高相关性分子集合进行了GSEA分析，旨在揭示这些分子集合在生物学通路和功能类别上的显著富集情况，从而进一步阐释FCN3在特定生物学过程中的作用机制。

### 1.11 免疫浸润分析

为深入解析FCN3高低表达组中的免疫浸润情况，利用ssGSEA（通过R包GSVA v1.46.0）和24种免疫细胞标志性基因，量化分析了样本中的免疫细胞浸润。基于ssGSEA算法对数据中FCN3和免疫浸润矩阵数据进行相关性分析，分析结果用ggplot2包进行棒棒糖图可视化免疫浸润情况。

### 1.12 免疫治疗反应与免疫检查点、免疫刺激分子及MHC分子

运用肿瘤免疫功能障碍与排除（Tumor Immune Dysfunction and Exclusion, TIDE）评分系统，对高低表达组患者的免疫逃逸特征展开评估。采用ESTIMATE算法（R包ESTIMATE）评估LUSC样本的基质和免疫成分。这些方法共同提供了多角度的免疫浸润分析，有助于深入理解FCN3在LUSC免疫调节中的作用。FCN3高、低表达组在免疫相关基因（包括免疫检查点基因、免疫刺激分子及MHC分子）表达上的差异，旨在揭示FCN3与免疫调节机制的潜在联系。

### 1.13 药物敏感性分析

使用R包oncPredict在癌症药物敏感性基因组学中获得药物的半数抑制浓度（half-maximal inhibitory concentration, IC_50_）值。通过斯皮尔曼分析药物IC_50_值与FCN3高低表达之间的相关性。绘制柱状图，比较FCN3高表达和低表达患者之间的IC_50_差异。

### 1.14 细胞培养

LUSC细胞（H226、SK-MES-1）和正常支气管上皮细胞（BEAS-2B）来自中国科学院上海细胞库（Shanghai Cell Bank, Chinese Academy of Sciences）。这些细胞在RPMI-1640培养基（Gibco）中培养，培养基中添加胎牛血清（FBS，来自新西兰）、100 IU/mL青霉素和10 µg/mL链霉素（Gibco）。所有细胞均在37 °C和5% CO_2_条件下培养。

实时荧光定量聚合酶链式反应（quantitative real-time polymerase chain reaction, RT-PCR）用Trizol试剂（Termo Fisher，16096020，美国）提取肺正常上皮及LUSC细胞系的总RNA。利用PrimeScript^TM^ RT Master Mix（Takara）对提取的RNA进行反转录。使用AceQ qPCR SYBR Green Master Mix（Vazyme，中国南京）进行qRT-PCR处理。然后获得Ct值，并采用2^-∆∆Ct^法计算目的基因的相对mRNA表达量。引物序列如下：FCN3 F：5′-TTAATGGTAACCGTACTTTCGCC-3′，R：5′-TGGTCAGCGTCATAGGTGGTA-3′。蛋白免疫印记收获细胞，用PBS冲洗后得到细胞沉淀。细胞裂解液经放射免疫共沉淀分析缓冲液配置，并在细胞沉淀中加入PMSF以获得细胞蛋白。蛋白样品经十二烷基硫酸钠-聚丙烯酰胺凝胶电泳分离后转移到硝酸纤维素膜上，然后用5%脱脂奶封闭2 h，然后在4 °C孵育抗体过夜。第二天，用TBST冲洗一抗，室温孵育二抗2 h，最后曝光。

### 1.15 统计学分析

所有数据处理与统计分析均借助R软件（版本4.1.2）完成。为评估两组间的生存率差异，采用Kaplan-Meier生存曲线、Cox比例风险回归模型及Log-rank检验，所有生存曲线均由R包survminer（版本4.1.2）绘制。在数据可视化方面，使用R包ggplot2（版本4.1.2）展示数据，并利用R包survival（版本4.1.2）计算预后评分。此外，通过R包pheatmap（版本4.1.2）创建热图。对于定量数据的差异分析，根据数据的分布情况选择合适的统计检验方法，对于符合正态分布的变量，采用双尾t检验或单因素方差分析来进行比较；对于非正态分布的数据，则选用Wilcoxon秩和检验来评估其差异是否具有统计学意义。计数资料组间比较采用卡方检验。相关性分析的统计方法选择Spearman等级相关。在本研究中，所有统计分析均通过R软件完成，并设定P<0.05为差异具有统计学意义的显著性水平。

## 2 结果

### 2.1 FCN3泛癌分析

对FCN3基因进行了泛癌分析，以揭示其在不同肿瘤类型中的表达模式及其预后影响，以及其与免疫微环境的潜在关联。结果显示，FCN3在多种癌症类型中均表现出显著的差异表达，其中在肾透明细胞癌（kidney renal clear cell carcinoma, KIRC）、弥漫性大B细胞淋巴瘤（lymphoid neoplasm diffuse large B-cell lymphoma, DLBC）、多形性胶质母细胞瘤（glioblastoma multiforme, GBM）、头颈鳞状细胞癌（head and neck squamous cell carcinoma, HNSC）肿瘤组织中的表达水平显著高于正常组织，而在肾上腺皮质癌、乳腺浸润癌、肺腺癌（lung adenocarcinoma, LUAD）、LUSC、肝细胞癌（liver hepatocellular carcinoma, LIHC）、胆管癌（cholangiocarcinoma, CHOL）等肿瘤中显著下调（P<0.0001）（图1A）。免疫浸润分析结果表明，FCN3的表达水平与多种癌症类型中免疫细胞的丰度（如T细胞和巨噬细胞）存在显著相关性，在CHOL和胸腺癌（thymoma, THYM）中，FCN3与CD8 T细胞的表达呈显著负相关，而在LUAD和LUSC中则呈显著正相关（图1B）。此外，研究发现FCN3的表达水平与LIHC、间皮瘤（mesothelioma, MESO）、GBM、LUSC、脑低级别胶质瘤（brain lower grade glioma, LGG）、KIRC、皮肤黑色素瘤（skin cutaneous melanoma, SKCM）等肿瘤的OS显著相关（P<0.05）（图1C）；与GBM、SKCM、LGG、KIRC肿瘤的DSS显著相关（P<0.05）（图1D）。以上研究结果表明FCN3可能作为这些肿瘤预后评估的一个重要生物标志物。以上研究结果表明，FCN3的表达水平在KIRC、LGG、GBM、KIRP、LIHC、MESO等肿瘤的预后评估中具有重要临床价值，且在不同癌症类型中的免疫浸润模式和生存期预后表现出显著的相关性，提示FCN3可作为多种类肿瘤的潜在预后生物标志物。

**图1:**
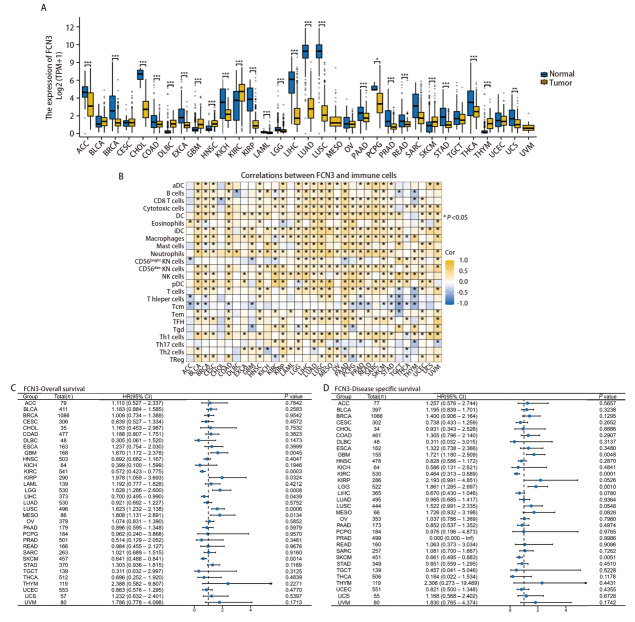
FCN3的泛癌分析。A：FCN3在不同类型肿瘤中的差异表达，FCN3在LUSC中低表达；B：FCN3在不同肿瘤中的免疫浸润情况；C：森林图可视化展示FCN3表达对OS的影响；D：森林图可视化展示FCN3表达对DSS的影响。*P<0.05，**P<0.01，***P<0.001。

### 2.2 基线特征分析

表1共纳入393例肺鳞癌患者，其中 FCN3低表达组192例，高表达组201例（表1）。临床病理特征方面，FCN3高表达组T1期患者比例显著高于低表达组（27.4% vs 16.1%, P=0.027），而N分期（P=0.165）和M分期（P=0.239）无显著差异。女性比例在FCN3高表达组显著升高（33.8% vs 17.2%, P<0.001），年龄（P=0.955）和吸烟史（P=0.220）在两组间无明显差异。PDCD1高表达在FCN3高表达组中更常见（61.7% vs 37.5%, P<0.001），CD274高表达亦呈现类似趋势（55.2% vs 45.3%, P=0.049）。结果提示FCN3高表达组在部分临床病理特征上表现出显著差异，尤其在肿瘤分期、性别比例以及PDCD1和CD274的表达上，提示FCN3的高表达可能与LUSC患者的临床特征存在一定关联。

**表1:** FCN3高表达组和低表达组的LUSC患者的临床病理特征

Characteristics	Low expression (n=192)	High expression (n=201)	P value
Pathologic T stage			0.027
T1	31 (16.1%)	55 (27.4%)	
T2	124 (64.6%)	113 (56.2%)	
T3&T4	37 (19.3%)	33 (16.4%)	
Pathologic N stage			0.165
N0	114 (59.4%)	136 (67.7%)	
N1	60 (31.3%)	46 (22.9%)	
N2 & N3	18 (9.3%)	19 (9.4%)	
Pathologic M stage			0.239
M0	191 (99.5%)	196 (97.5%)	
M1	1 (0.5%)	5 (2.5%)	
Gender			<0.001
Female	33 (17.2%)	68 (33.8%)	
Male	159 (82.8%)	133 (66.2%)	
Age (yr)			0.955
≤65	76 (39.6%)	79 (39.3%)	
>65	116 (60.4%)	122 (60.7%)	
Smoking history			0.220
No	5 (2.6%)	10 (5.0%)	
Yes	187 (97.4%)	191 (95.0%)	
PDCD1			<0.001
Low	120 (62.5%)	77 (38.3%)	
High	72 (37.5%)	124 (61.7%)	
CD274			0.049
Low	105 (54.7%)	90 (44.8%)	
High	87 (45.3%)	111 (55.2%)	

Data acquisition: Download and sort out RNAseq data of STAR process of TCGA-LUSC project from TCGA database and extract TPM format. Data filtering strategy: remove normal+remove no clinical information. Missing data were handled by excluding samples with missing values for any variable. Data processing method: log2(value+1). TCGA: The Cancer Genome Atlas.

### 2.3 表征和评估FCN3在LUSC中的表达水平及对患者预后的影响

在LUSC组织中FCN3的表达水平显著低于正常组织（图2A），基于TCGA数据库LUSC样本评估FCN3表达水平及与患者预后的相关性，以FCN3高低表达分组构建Kaplan-Meier生存曲线。并依据肿瘤原发灶-淋巴结-转移（tumor-node-metastasis, TNM）分期系统，对患者进行了分类，针对不同T、N分期以及综合分期的患者子集分别绘制了Kaplan-Meier生存曲线。此外，考虑到性别因素可能在疾病进展和预后中扮演重要角色，我们特别设立了男性和女性亚组，分别探究FCN3表达水平在这两个群体中的生存影响。研究结果显示，与FCN3低表达组患者相比，FCN3高表达患者的OS明显较差（图2B）。女性亚组的Kaplan-Meier结果不及男性亚组结果显著（图2C）。其他根据分期的亚组结果均可见到高表达组的OS较短（图2D-2F），将FCN3表达量、T分期、病理分期作为构建LUSC患者1、3、5年生存率的Nomogram模型指标（图3A），列线图模型的一致性指数（C-index）：0.621（0.600-0.642），受试者工作特征（receiver operating characteristic, ROC）曲线分析1、3、5年曲线下面积（area under the curve, AUC）值为0.636、0.677、0.680（图3B）。预后校准曲线结果1、3、5年OS校正曲线与理想参考线接近。模型预测一致性良好（图3C）。这些结果表明，FCN3在LUSC中的高表达与较差的预后相关，且能有效预测LUSC患者的1、3、5年生存率，具有潜在预后价值。

**图2:**
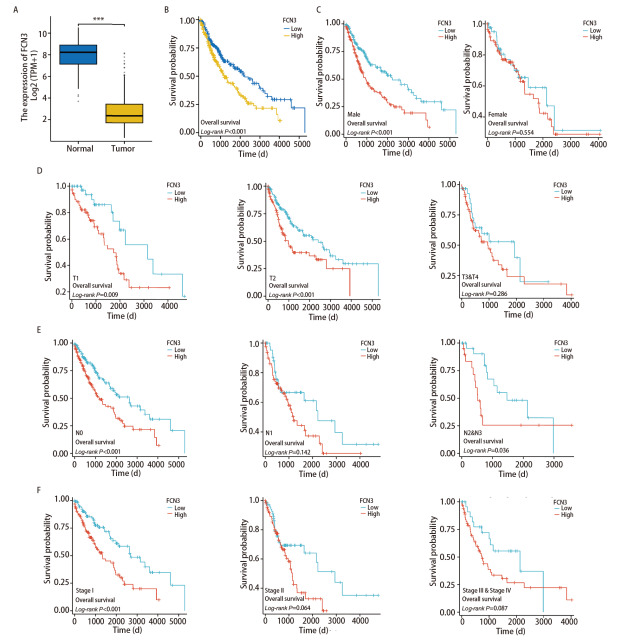
FCN3在LUSC中的预后和ROC曲线分析以及FCN3表达与LUSC不同临床特征的相关性。A：FCN3在LUSC的表达情况；B：根据FCN3表达水平绘制的LUSC患者的Kaplan-Meier图；C：在不同性别中FCN3表达差异；D-F：在肿瘤的TNM分期中，FCN3与 T（肿瘤）和N（淋巴结）和 M（转移）均相关。***P<0.05。

**图3:**
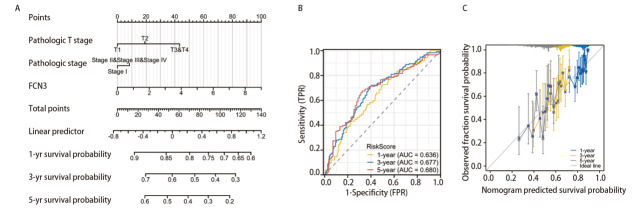
构建LUSC患者1、3、5年生存预后Nomogram模型。A：构建Nomogram模型；B：用于预测LUSC中1、3和5年OS列线图的ROC曲线; C：用于预测LUSC中1、3和5年OS的列线图校准曲线。

### 2.4 FCN3表达与LUSC基因突变模式的关联分析

对FCN3高低表达组的TMB分析结果显示，FCN3高表达组的TMB显著低于低表达组（图4A），且该差异在统计上具有显著性（P<0.001）。低表达组中突变频率较高的基因包括TP53、CSMD3、TTN、MUC16、RYR2 等。其中，TP53突变率最高，其余基因在该组中也呈现较高突变频率，反映出该组具有较高的TMB（98.71%）（图4B）。高表达组的突变模式虽然主要突变基因的突变频率总体与低表达组相似（97.38%），但整体突变负荷较低，部分基因的突变频率在高表达组中有所下降，这与TMB的整体降低趋势一致（图4C）。以上结果表明，FCN3高表达组的突变负荷较低。两组主要突变基因一致，但TMB的下降可能影响基因组稳定性和突变积累过程。

**图4:**
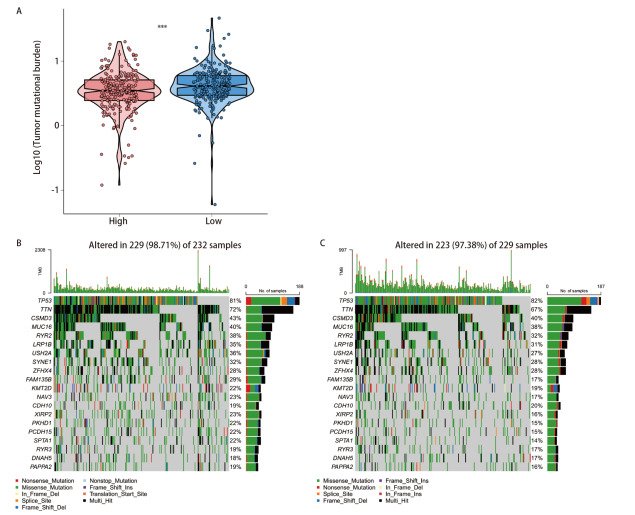
FCN3的高低表达在LUSC中的体细胞突变分析。A：FCN3高低表达组的TMB差异；B：低表达组突变频率最高的前20个基因；C：高表达组突变频率最高的前20个基因。***P<0.001。

### 2.5 FCN3高低表达组差异基因及其在LUSC中的功能与信号通路分析

确定FCN3在LUSC中的异常表达扰动的枢纽基因，探究其参与调控的生物学过程和关键的信号通路，表征FCN3在LUSC中可能的功能。我们首先通过Rggpolt2包可视化为火山图及热图（图5A-5B），再通过单基因差异分析，并对高、低表达组进行比较分析，共确定了27个差异表达基因（|log2Fold Change|>2，FDR矫正后的P值<0.05）。在High_expression样本中，27个基因上调，0个基因下调。为了研究关键枢纽基因的生物学功能，我们进行了GO功能和KEGG通路富集分析。富集结果包括：体液免疫反应、体液免疫反应的调节、抗菌体液反应、白细胞迁移、急性炎症反应、白细胞趋化等（图5C）。GSEA分析结果包括：表面活性剂代谢通路、细胞外基质相关通路、代谢性疾病通路、固有免疫系统通路（图5D）。以上结果表明FCN3经过补体激活参与先天免疫反应。

**图5:**
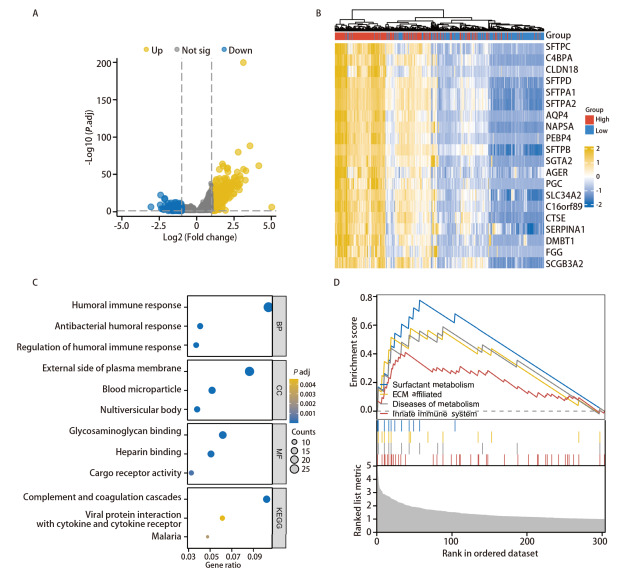
鉴定LUSC中与FCN3功能相关的枢纽基因，并进行GO/KEGG富集分析和GSEA分析。A：火山图展示TCGA数据库分析的LUSC中与FCN3表达水平相关的DEGs；B：热图可视化展示FCN3表达的差异基因；C：GO和KEGG通路富集分析；D：对DEGs基因GSEA富集分析，这些基因在多个生物学通路中显著富集。

### 2.6 FCN3相关基因的功能富集分析及其在免疫反应中的作用

此外，通过Spearman相关系数分析，筛选与FCN3基因表达显著相关的mRNA分子（|r|>0.5, P<0.05），探究其参与调控的生物学过程和关键的信号通路，进一步表征FCN3在LUSC中可能的功能。分析共识别出260个高相关性基因（图6A）。随后，我们构建260个基因的PPI网络（图6B），并对FCN3高相关性基因进行GSEA分析后，研究结果发现，基因集在多个生物学通路中显著富集，主要包括： T细胞受体复合物、免疫球蛋白复合物、抗原结合、免疫受体活性、髓系白细胞激活、由白细胞介导的免疫反应、由淋巴细胞介导的免疫反应（图6C），而NSE为负值的通路包括：DNA复制过程涉及DNA的合成和复制、DNA构象变化、DNA复制过程（图6D）。以上分析结果表明FCN3不仅参与了T细胞、B细胞以及髓系白细胞的免疫激活过程，还增强了由淋巴细胞和白细胞介导的适应性免疫反应。

**图6:**
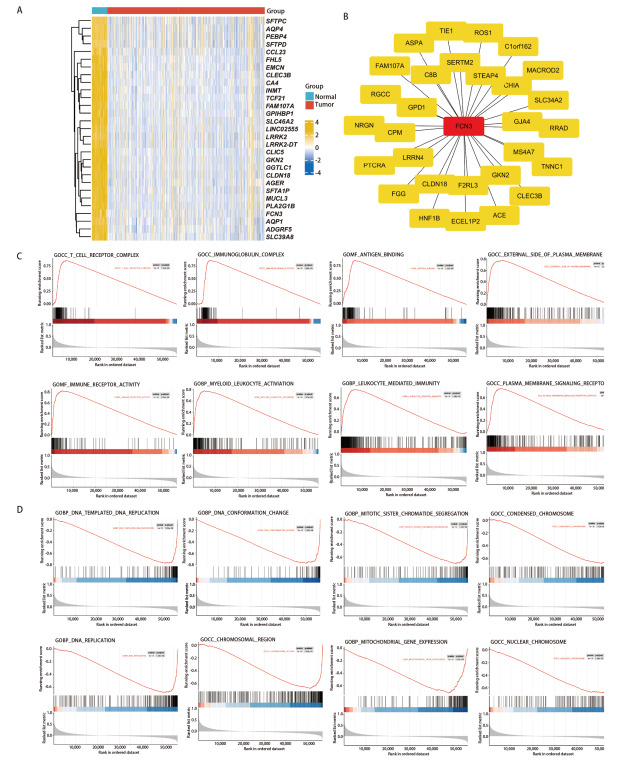
FCN3高相关性基因鉴定和通路富集。A：热图可视化与FCN3基因表达显著相关的分子；B：使用STRING数据库构建FCN3的蛋白关系网络；C：FCN3高相关性基因的GSEA富集分析（NSE为正值）；D：FCN3高相关性基因的GSEA富集分析（NSE为负值）。

### 2.7 FCN3表达与免疫细胞浸润和免疫细胞功能的关联

FCN3高表达组在ESTIMATE免疫评分（StromalScore、ImmuneScore和ESTIMATEScore）中均显著高于低表达组（P<0.001）（图7A），提示FCN3高表达患者的肿瘤微环境（tumor mircroenvironment, TME）中基质成分和免疫细胞浸润更为丰富。进一步通过TIDE评分发现，FCN3高表达组的Dysfunction评分显著升高（P<0.001）（图7B），表明其TME中可能存在更强的免疫功能障碍，而低表达组则可能表现出更强的免疫排斥反应。此外，免疫浸润分析结果显示，24种免疫细胞中多数（如CD8 T细胞、DC、巨噬细胞）在FCN3高表达组的丰度显著增高（图7C-7D），提示这些免疫细胞在高表达组中可能处于更高的浸润或激活状态。这些结果表明，FCN3高表达与TME中基质成分和免疫细胞浸润的显著增加有关，可能通过调控免疫细胞功能参与肿瘤的免疫逃逸和进展。

**图7:**
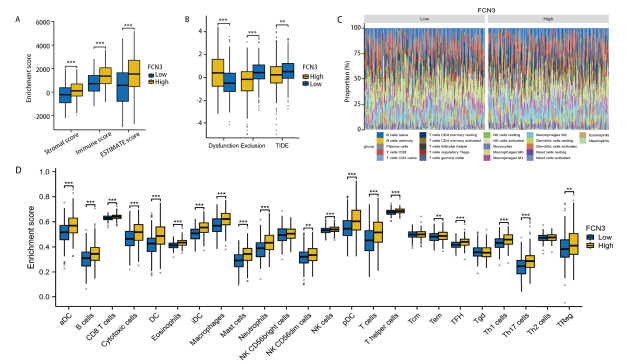
FCN3的表达与免疫浸润和免疫治疗反应的相关性。A：采用ESTIMATE算法评估LUSC样本的免疫浸润情况；B：TIDE免疫评分与FCN3表达的关系；C，D：利用ssGSEA可视化24种免疫细胞标志性基因，展示FCN3高低表达组中的免疫浸润情况。**P<0.01，***P<0.001。

### 2.8 FCN3对免疫检查点、免疫刺激分子和MHC分子表达的影响

我们在FCN3高低表达组间对免疫检查点基因的表达进行比较，结果发现多种免疫检查点在两组间存在显著差异，23种免疫检查点分子中，除ADORA2A、TGFB1、TGFBR1、VTCN1外，均在FCN3高表达组中表达水平显著升高（图8A），且与FCN3表达呈正相关（图8B）。另外，43种免疫刺激分子在两组间的比较结果显示IL6 、IL6R、TNFSF14等多个分子的表达在高FCN3组中显著升高（图9A），且部分分子的表达与FCN3呈显著正相关（图9B）。对21种MHC类分子在FCN3高低表达组中的表达分析结果提示：全部21种MHC类分子的基因表达均显著上调（图10A）。且MHC类分子的表达与FCN3均呈显著正相关（图10B）。以上结果表明，FCN3的高表达与免疫检查点分子、免疫刺激分子及MHC类分子的表达水平呈显著正相关，提示FCN3可能在肿瘤免疫微环境中发挥重要调控作用。这些发现为进一步探索FCN3在肿瘤免疫逃逸和免疫治疗中的潜在机制提供了重要线索。

**图8:**
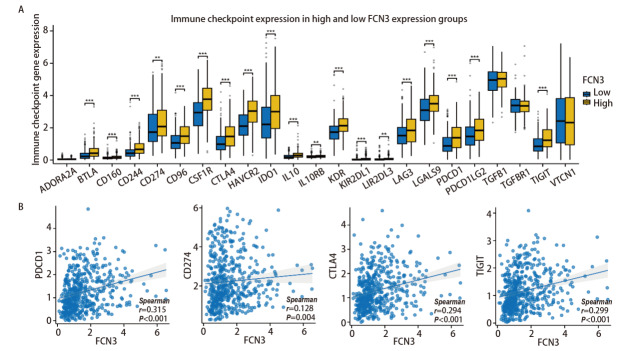
LUSC中FCN3的表达与免疫检查点分子相关性。A：LUSC中FCN3高低表达组中免疫检查点分子表达的差异；B：FCN3与PDCD1、CD274、CTLA4和TIGIT表达量的相关性分析。**P<0.01，***P<0.001。

**图9:**
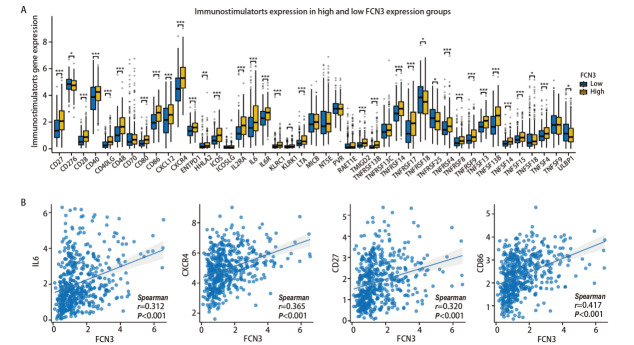
LUSC中FCN3的表达与免疫刺激分子相关性。A：LUSC中FCN3高低表达组中免疫刺激分子表达的差异；B：FCN3与IL6、CXCR4、CD27和CD86表达量的相关性分析。*P<0.05，**P<0.01，***P<0.001。

**图10:**
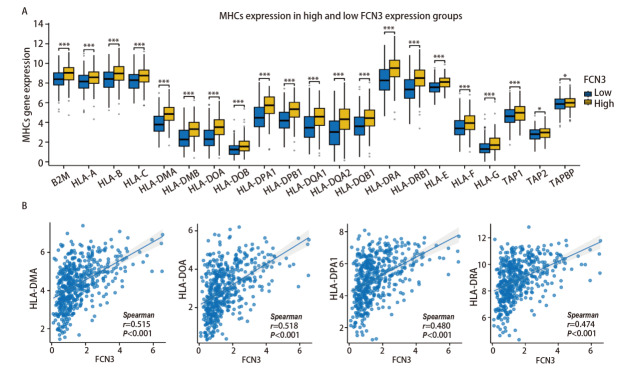
LUSC中FCN3的表达与MHC分子相关性。A：LUSC中FCN3高低表达组中MHC分子表达的差异；B：FCN3与MHC-DMA、HLA-DOA、HLA-DPA1和HLA-DRA表达量的相关性分析。*P<0.05，***P<0.001。

### 2.9 FCN3表达与LUSC化疗药物敏感性的关联

进一步在高低表达组之间进行了药物敏感性分析，以评估对这两组潜在有效的主要化疗药物。结果表明，相较于高表达组，低表达组的样本对紫杉醇（Paclitaxel）、多西他赛（Docetaxel）、长春瑞滨（Vinorelbine）、Sepantronium bromide_1941、Dactinomycin_1911、Daporinad_1248、Eg5_9814_1712的反应更为敏感。而相比低表达组，高表达组的样本对AZD8055_1059和Topotecan_1808（拓扑替康）的反应则更为敏感（y轴值越小，说明药物敏感性越高）（图11）。我们的研究结果显示，FCN3高表达与几种广泛使用的癌症药物（包括紫杉醇、多西他赛、长春瑞滨）的敏感性降低有关。这些发现表明了FCN3在化疗耐药中的作用及其作为治疗靶点的潜力。

**图11:**
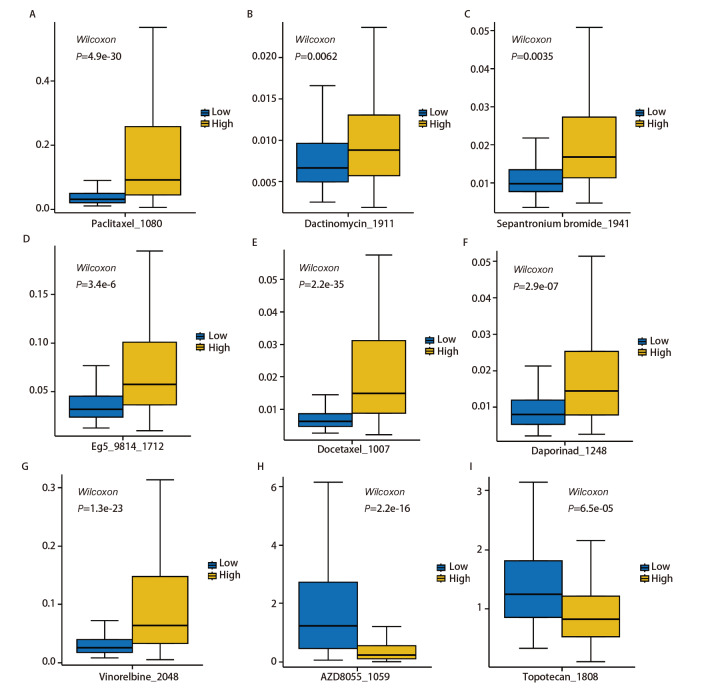
FCN3表达与药物敏感性的相关性分析（P<0.05）。A：紫杉醇； B：放线菌素D；C：Sepantronium bromide；D：Eg5_9814；E：多西他赛；F：达珀利奈；G：长春瑞滨；H：AZD8055；I：托泊替康。

### 2.10 体外实验与数据库分析验证FCN3表达

为进一步验证数据分析的准确性，我们通过体外实验验证了FCN3在LUSC中的表达情况。与正常支气管上皮细胞相比，LUSC细胞系H226和SK-MES-1的mRNA水平明显降低（图12A），这与前面数据库得到的信息一致。随后又通过HPA公共数据库验证了在LUSC组织中，正常组织中FCN3的表达明显高于肿瘤组织（图12B）。最后，蛋白免疫印迹实验也在蛋白水平验证了前面的结果，FCN3在LUSC中低表达（图12C），也证实了文中一系列的数据分析是有意义的，其有作为LUSC中独立的预后标志物的潜力。

**图12:**
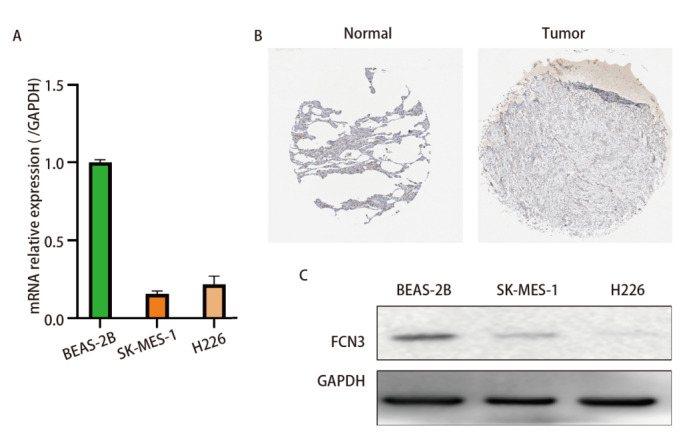
体外实验验证FCN3在LUSC的表达。A：LUSC细胞系中FCN3的mRNA表达水平；B：FCN3在正常肺组织和LUSC中的免疫组化染色结果。染色使用了抗体CAB025945，展示了FCN3在这两种组织中的表达差异。图片来源：Human Protein Atlas，访问链接： https://www.proteinatlas.org/；C：LUSC细胞系中FCN3的蛋白表达水平。

## 3 讨论

在全球范围内，肺癌的发病率和致死率仍然居高不下，是一直存在的公共威胁。然而，LUSC的突变情况较为复杂，因此可用的靶向药物十分有限。免疫治疗已成为LUSC的热点治疗方式，但由于个体免疫微环境的差异，其效果存在显著差异^[15]^。开发有效的生物标志物已成为LUSC免疫疗法的迫切需求。

通过泛癌分析，我们发现FCN3在多种癌症类型中的表达有着显著差异，且与多种癌症的OS、DSS密切关联，在LUSC中，FCN3的表达增高与肺鳞癌患者较差预后密切关联。

有研究^[16]^表明，TMB与肺癌免疫治疗反应的关系，并强调了TP53等基因在高TMB的肺癌患者中突变频率可能提高肿瘤的免疫原性，与免疫逃逸机制相关。我们对FCN3高低表达组间TMB分析结果提示FCN3高表达组的较低TMB可能意味着该组肿瘤的新生抗原较少，这可能影响肿瘤细胞被免疫系统识别的能力。FCN3可能通过调控基因组稳定性和减少突变负荷，在肿瘤免疫逃逸中发挥作用，影响免疫微环境，降低免疫系统的识别和攻击能力。

我们进一步通过对LUSC中FCN3高低表达组进行了ESTIMATE评分，分析肿瘤样本中免疫细胞和间质细胞的基因表达，推测TME的免疫状态。结果表明，FCN3的高表达可能通过上调TME中的免疫细胞浸润和基质成分，参与免疫反应的调节。此外，FCN3还通过激活补体途径参与适应性免疫反应。而在TIDE评分结果中，FCN3高表达组的Dysfunction（T细胞功能障碍）显著高分，提示FCN3高表达组的免疫功能障碍程度较高，TIDE评分结果反映了各种免疫逃逸机制的潜在参与。这可能意味着这些患者的肿瘤免疫抑制作用较强，使得肿瘤逃避免疫系统的识别和清除。

已有研究^[17]^表明FCN3通过其纤维蛋白原样结构域与病原体表面的碳水化合物结合，激活甘露糖结合凝集素相关丝氨酸蛋白酶，从而启动补体途径。TME中的补体激活能通过减少细胞凋亡来维持肿瘤细胞的生存，还通过过敏毒素受体信号转导和膜攻击复合物的组装，进一步促进肿瘤细胞的去分化、增殖和迁移^[18]^。为进一步挖掘这些免疫微环境差异背后的关键分子通路及其免疫调控机制。我们基于两种方法获得不同的分子集合分别做了GSEA分析。首先对FCN3高低表达分组进行差异分析，对显著差异的分子进行GSEA分析，结果提示：FCN3参与体液免疫反应及其调节、固有免疫、病原清除和补体激活、白细胞迁移、趋化及急性炎症反应。在LUSC中FCN3可能通过其参与的补体激活、趋化因子网络和炎症微环境影响肿瘤进展。另外，我们经过相关性分析获得FCN3高相关性分子集合（|r|>0.5, P<0.05）并进行GSEA分析，结果提示FCN3可能间接参与T细胞和B细胞介导的适应性免疫反应、可能调控免疫细胞表面的受体活性，且可能参与抗体和抗原结合的识别与调节。其中髓系/淋巴细胞介导的免疫反应展示了FCN3与固有免疫（髓系白细胞激活）和适应性免疫（淋巴细胞介导的免疫反应）的联系。

已有研究^[19]^证明免疫检查点抑制剂已成为LUSC患者的有效治疗方法。基于免疫功能的显著关联，我们进一步关注FCN3是否可能通过调控免疫检查点分子的表达，参与肿瘤的免疫逃逸机制。结果发现多数免疫检查点分子如PDCD1、CTLA4等在FCN3高表达组中显著上调。免疫检查点分子与配体结合，抑制T细胞活化信号通路，包括：抑制T细胞受体下游信号通路^[20]^ ；降低T细胞增殖和效应分子的产生[如干扰素γ（interferon gamma, IFN-γ）和白细胞介素2（interleukin 2, IL-2）]^[21]^；增强T细胞衰竭状态^[22]^，进而促进免疫逃逸。这种广泛的显著差异结果提示FCN3可能通过调节多数免疫检查点的表达水平来影响肿瘤的免疫逃逸和患者的免疫治疗反应。同时也说明FCN3在免疫逃逸机制中的潜在作用。

然而，肿瘤免疫微环境的构建并非仅依赖于免疫检查点，免疫刺激分子在其中同样扮演重要角色，其不仅通过增强免疫细胞的功能促进抗肿瘤反应，同时在某些条件下也可能通过调控肿瘤周围的微环境细胞（如成纤维细胞和间质细胞）促进肿瘤的生长和转移^[23]^。本研究观察到FCN3高表达组中TNFSF分子和IL6R等免疫刺激因子的显著上调，提示其可能通过促进血管生成、激活JAK/STAT和PI3K/AKT信号通路，进一步参与TME的重塑。

研究^[24]^表明肿瘤细胞通过改变MHC-I分子的表达和功能，能够逃避免疫系统的监视和攻击，这不仅促进了肿瘤的进展，还可能降低癌症免疫治疗的效果。MHC-II在将抗原呈递给CD4 T细胞方面起着关键作用^[25]^，而CD4^+^ T细胞支持CD8^+^ T细胞活化、记忆T细胞的产生^[26]^。肿瘤特异性的MHC-II与癌症患者（包括接受免疫治疗的患者）的良好预后密切相关。我们将研究重点转向MHC分子，探索其在FCN3高低表达组中的表达差异，结果提示：与FCN3低表达组相比，高表达组中多个MHC类分子的基因表达显著上调，这提示FCN3与MHC类分子的表达之间可能存在正相关关系，例如，HLA-A、HLA-B、HLA-C、B2M、HLA-DMA、HLA-DRB1等基因的表达在FCN3高表达组中显著增加。

FCN3在肿瘤免疫领域中起着双重角色，FCN3上调免疫刺激分子和MHC I类和II类分子的表达识别，不仅可以激活T细胞从而增强抗肿瘤免疫反应，还可能降低免疫治疗效果。FCN3又通过上调多数免疫检查点的表达，弱化免疫系统的杀伤作用、促成肿瘤细胞的免疫逃避。结合高表达组的生存期显著较短这一事实，这两种免疫调节中，FCN3通过上调免疫检查点促癌免疫逃逸作用更加显著和重要。可以推测，在免疫微环境中免疫检查点分子表达较高的LUSC患者中，抑制FCN3可能与免疫检查点抑制剂产生协同作用。而且，根据我们的药物预测分析结果，抑制FCN3也可能改善对高表达组患者化疗耐药情况。

总之，本研究揭示了FCN3在LUSC中发挥着重要且复杂的免疫调节作用，不仅调控了免疫细胞的功能，更通过广泛的免疫检查点作用机制促进免疫逃逸。这些发现为FCN3成为治疗LUSC的潜在免疫治疗靶点提供了坚实的理论依据。然而，本研究尚存在一些局限性，尤其是缺乏深入的细胞和动物实验验证来进一步支持我们的结论。未来，我们将致力于开展更为详尽的实验研究，以全面评估FCN3在LUSC免疫治疗中的潜力和效果。
